# Burden, risk factors, and comorbidities of behavioural and emotional problems in Kenyan children: a population-based study

**DOI:** 10.1016/S2215-0366(16)30403-5

**Published:** 2017-02

**Authors:** Symon M Kariuki, Amina Abubakar, Martha Kombe, Michael Kazungu, Rachael Odhiambo, Alan Stein, Charles R J C Newton

**Affiliations:** a KEMRI-Wellcome Trust Collaborative Research Programme, Kilifi, Kenya; b Department of Public Health, Pwani University, Kilifi, Kenya; c Department of Psychiatry, University of Oxford, Oxford, UK

## Abstract

**Background:**

Three-quarters of the burden of mental health problems occurs in low-and-middle-income countries, but few epidemiological studies of these problems in preschool children from sub-Saharan Africa have been published. Behavioural and emotional problems often start in early childhood, and this might be particularly important in Africa, where the incidence of perinatal and early risk factors is high. We therefore aimed to estimate the prevalence and risk factors of behavioural and emotional problems in young children in a rural area on the Kenyan coast.

**Methods:**

We did a population-based epidemiological study to assess the burden of behavioural and emotional problems in preschool children and comorbidities in the Kilifi Health and Demographic Surveillance System (KHDSS, a database formed of the population under routine surveillance linked to admissions to Kilifi County Hospital). We used the Child Behaviour Checklist (CBCL) to assess behavioural and emotional problems. We then determined risk factors and medical comorbidities associated with behavioural and emotional problems. The strength of associations between the risk factors and the behavioural and emotional problems was estimated using generalised linear models, with appropriate distribution and link functions.

**Findings:**

3539 families were randomly selected from the KHDSS. Of these, 3273 children were assessed with CBCL. The prevalence of total behavioural and emotional problems was 13% (95% CI 12–14), for externalising problems was 10% (9–11), and for internalising problems was 22% (21–24). The most common CBCL syndrome was somatic problems (21%, 20–23), whereas the most common DSM-IV-oriented scale was anxiety problems (13%, 12–14). Factors associated with total problems included consumption of cassava (risk ratio 5·68, 95% CI 3·22–10·03), perinatal complications (4·34, 3·21–5·81), seizure disorders (2·90, 2·24–3·77), and house status (0·11, 0·08–0·14). Seizure disorders, burn marks, and respiratory problems were important comorbidities of behavioural and emotional problems.

**Interpretation:**

Behavioural and emotional problems are common in preschool children in this Kenyan rural area and are associated with preventable risk factors. Behavioural and emotional problems and associated comorbidities should be identified and addressed in young children.

**Funding:**

Wellcome Trust.

## Introduction

Behavioural and emotional problems are common in preschool children aged younger than 6 years in high-income countries.^1^ These problems in preschool children are classified into two broad categories: externalising problems such as aggressive behaviour and internalising problems such as emotional disturbances.^2^ Epidemiological and meta-analytic studies of behavioural and emotional problems in children often combine preschool estimates with those of older children, contributing to a scarcity of data in young children, particularly in Africa.^3^ Childhood behavioural and emotional problems increase the risk of mental health problems later in life,^4^ which could be prevented with early identification and management of these behavioural and emotional problems.

The prevalence of behavioural and emotional problems in high-income countries ranges from 3% to 40%.^5^ Estimates of behavioural and emotional problems from the Middle East, Asia, and South America range from 10% to 30%.^6^ Some of these low-and-middle-income studies were based on hospital samples,^6^ which are biased towards greater morbidity. No reliable epidemiological studies of behavioural and emotional problems in preschool children from Africa have been published, which affects planning for preventive and therapeutic interventions such as the Triple P-positive Parenting Programme for parents.^7^ Identification of behavioural and emotional problems in preschool children from low-and-middle-income countries is challenging because these children are developing rapidly, and few tools exist to assess these problems.^8^

Behavioural and emotional problems in high-income countries^9^ are associated with risk factors such as pregnancy and perinatal factors, social disadvantage, family factors, and environmental factors, which can be addressed through public health interventions and social policy change. Most of these risk factors are very common in Africa, where they could contribute to the burden of behavioural and emotional problems in young children. Behavioural and emotional problems co-occur with febrile seizures (in 20% of young children),^10^ malnutrition, and sleep problems.^11^ These comorbid conditions have not been examined in African countries.

**Research in context**

**Evidence before this study**

We searched PubMed for all English articles published up to Aug 31, 2016, using the terms “behavioural and emotional problems”, “mental health problems/disorders”, “externalising problems/disorders”, “internalising problems/disorders”, and “preschool/young children”, “infants”. Most studies of behavioural and emotional problems in preschool children were from high-income countries, with few from low-and-middle-income countries of the Middle East, Asia, and South America. There were no studies from Africa. Most studies were done after the year 2000 suggesting a recognition of the need to understand behavioural and emotional problems in preschool children. The prevalence ranged from 3% to 40% in high-income countries, and 10% to 30% in low-and-middle-income countries, although some studies from low-and-middle-income countries were hospital based. The high-income country studies identified antenatal or perinatal complications, social disadvantages, environmental factors, and family factors as the risk factors for behavioural and emotional problems. The commonest comorbidities of behavioural and emotional problems were sleep problems, upper respiratory problems, and seizures.

**Added value of this study**

This study provides robust evidence of the high burden of behavioural emotional problems in preschool children from a rural area on the Kenyan coast, and identifies seizure disorders, perinatal complications, and lifestyle factors such as consumption of cassava and soil as the most important risk factors. Most of these risk factors are easily preventable. Medical comorbidities such as seizures, burns, and respiratory infections were more frequent in those with behavioural and emotional problems than those without.

**Implications of all the available evidence**

Preschool children experience a high burden of behavioural and emotional problems similarly to older children; these problems should be assessed and addressed, alongside the associated medical comorbidities. Control of causes and risk factors for seizure disorders might reduce the burden of behavioural problems in this rural area of Kenya. The mental health problems should be addressed before children go to school.

We did an epidemiological study to estimate the prevalence and risk factors of behavioural and emotional problems in young children in a rural area on the Kenyan coast. Clinical examination was done on a random sample to identify the medical comorbidities of behavioural and emotional problems.

## Methods

### Study design and participants

This study was done within the Kilifi Health and Demographic Surveillance System (KHDSS), which is located in Kilifi County, about 60 km north of Mombasa city.^12^ The KHDSS is both an area (divided into enumeration zones under regular surveillance) and a database (formed of the population under routine surveillance linked to admissions to Kilifi County Hospital). The KHDSS has a population of about 280 000 residents who are predominantly of the Mijikenda tribe and has an estimated 50 000 children aged 1–6 years. The KHDSS has a northern and southern region covering an area of 891 km^2^. Epilepsy and neurodevelopmental clinics at Kilifi County Hospital provide therapeutic interventions and counselling services. Screening in stage 1 was done by trained fieldworkers who read out the content of the questionnaires to the parents owing to low literacy levels in this area, taking short breaks every 10 min. The three questionnaires (for risk factors, behavioural and emotional problems, and seizures) in stage 1 were administered in a random order. A random sample of those with and without behavioural and emotional problems predetermined through a sample calculation was invited in stage 2 for a clinical evaluation study to determine medical comorbidities. This study was approved by the Scientific and Ethics Review Unit of the Kenya Medical Research Institute and written informed consent was obtained from parents or carers of children participating in the study.

### Procedures

Behavioural and emotional problems were assessed in stage 1 with the Child Behaviour Checklist (CBCL), which was adapted and piloted in the local population and languages.^13^ The CBCL is used in children aged 1·5–5·5 years,^2^ and has been applied on 1–6-year-old children; it identifies seven syndromes and five DSM-IV-oriented scales.^14^ The CBCL has acceptable psychometric properties on a sample of Kenyan preschool children in this area.^13^

The CBCL items had an internal consistency Cronbach's α of 0·95, and inter-informant agreement (Pearson's correlation coefficient, *r*>0·80), test–retest reliability (*r*=0·76), and the fit index of the seven-CBCL syndromes (eg, root mean square error of approximation <0·05) were within acceptable ranges.^13^ Because of the literacy challenges in this area, CBCL questions were read out to the respondents (parents or guardians) by trained neuropsychological assessors fluent in the local languages. The language of administration was primarily Kiswahili (lingua franca), but Giriama was also used for a few respondents who could not comprehend Kiswahili. We used a systematic approach of translation and adaptation of the tools. The initial translation was done by two independent translators fluent in the original language (English) and the target language (Kiswahili and Giriama). These translations were then back translated into English by two independent translators and inconsistencies were resolved.

The scoring system used and the grouping of the CBCL items into syndromes and externalising and internalising subscales followed recommendations by the Achenbach System of Empirically Based Assessments.^2^ The total score for the CBCL was formed by summing ratings from all of the 99 items. Items that formed the seven syndromes of the CBCL, externalising and internalising scores, and the DSM-oriented scales are outlined in the appendix.

The 90th percentile of the CBCL scores was used as the cutoff according to recommendations from the developers of the tool, and produced cutoff scores similar to those applied in children in the USA.^15^ These cutoffs were piloted and found to discriminate between children with and without adverse perinatal events.^13^ Parents and carers of children assessed with the CBCL were interviewed using a parental risk factor questionnaire. The risk factor questionnaire consisted of the following items: maternal sociodemographic information such as employment, schooling, religion, and ethnicity; pregnancy and perinatal histories; socioeconomic status indicators such as housing status; medical histories such as seizure disorders or other chronic illnesses; and child health and nutrition habits, such as food types consumed, eating soil, and snoring at night. A thorough literature search informed the choice of risk factors included in the questionnaire.

About 20% of the children were invited to take part in stage 2 of the study. One clinician saw 243 children with CBCL scores of more than 60 and 377 children with CBCL scores of less than 60. The children were randomly selected from those screened in stage 1 using the RAND command of MySQL (Oracle Corp, USA). The clinician who was blinded to the screening status in stage 1 asked questions about the history of seizures to determine whether the seizures were acute seizures or epilepsy. The clinician was blinded to the status of the children screening positive for seizures in stage 1 in the community. The clinician did a clinical examination to assess for gross and fine motor deficits, sensory function, abnormal limb activity, cognitive or mental status, cranial nerve function, sensory ability, and skin integrity. Anthropometric measures of the child were also taken and included head circumference, mid-upper arm circumference, height, and weight.

Abnormal pregnancy was defined as premature or prolonged labour, post-dated pregnancy, pre-eclampsia, eclampsia, or any other health problems during pregnancy.^16^ Adverse perinatal events were defined as delays in crying, breathing, and breastfeeding after birth. Seizure disorders included both epilepsy and febrile acute seizures, with febrile seizures defined as seizures associated with a febrile illness or fever in those younger than 6 years, and epilepsy as a history of two unprovoked seizures.^17^ Intellectual disability was assessed by a clinician observing young children who had problems performing the standardised test of a locally adapted developmental inventory.^18^ Malnutrition was defined as a weight-for-age *z* score value of −2 or lower or a mid-upper arm circumference less than 11·5 cm. Sensory function was considered impaired if a child could not localise touch from cotton wool or a painful stimulus. Motor impairments were defined as an inability to hold toys and walk or sit upright if of an appropriate age.

### Statistical analysis

We estimated that screening approximately 3500 children aged between 1 and 6 years, randomly selected from a surveillance database of 50 000 children using RAND command would be sufficient to identify psychopathology with a precision of 1%. We assessed whether the CBCL scores had a normal distribution by plotting quintile, Kernel density (of predicted regression residuals), and histogram plots (appendix) on raw and (natural) log-transformed scores.

Prevalence of behavioural problems was computed first as a probability (where those with CBCL problems are treated as positive and those without CBCL problems as negative), applying the inverse logit function to the intercept coefficient of a logistic regression model to provide outcome probabilities on the logit (log odds) scale. The probability was then multiplied by 100 to obtain the prevalence. Prevalence estimates stratified by age group and sex were computed, fitting fractional polynomial equations to smooth the prevalence by age.

Risk factors associated with behavioural and emotional problems were determined using log binomial regression models implemented in a generalised linear model, with robust variance-covariance matrix of the estimators. β coefficients for each risk factor above were then computed with log-transformed CBCL scores as the dependent variable using a generalised linear model that assumes a normal distribution and has an identity link function, and a robust variance–covariance matrix. We built penultimate models that accounted for child factors (age, sex, schooling, and region of residence) and final models that accounted for both child factors and maternal factors (age, marital status, economic or employment status, education level, ethnicity, and religion). A test for linear trend was performed for risk factors categorised into three or more levels using likelihood ratio tests. Age for the child and mother were entered into the risk factor models as first degree fractional polynomials. We measured associations between discrete variables using Pearson's χ^2^ tests, or Fisher's exact tests, where the number of observations in a cell was less than five. Student's *t* test or the Mann-Whitney *U* test were used to compare the distribution of behavioural and emotional scores. Internal consistency was computed using the cialpha command in Stata. A p value of 0·05 or less was considered significant for exploratory comparisons, whereas associations were significant if the lower CIs did not include 1. All analyses were done with Stata, version 13.

### Role of the funding source

The funders of the study had no role in study design, data collection, data analysis, data interpretation, or the writing of the report. The corresponding author had full access to all the data in the study and had final responsibility for the decision to submit for publication.

## Results

**Figure 1:**
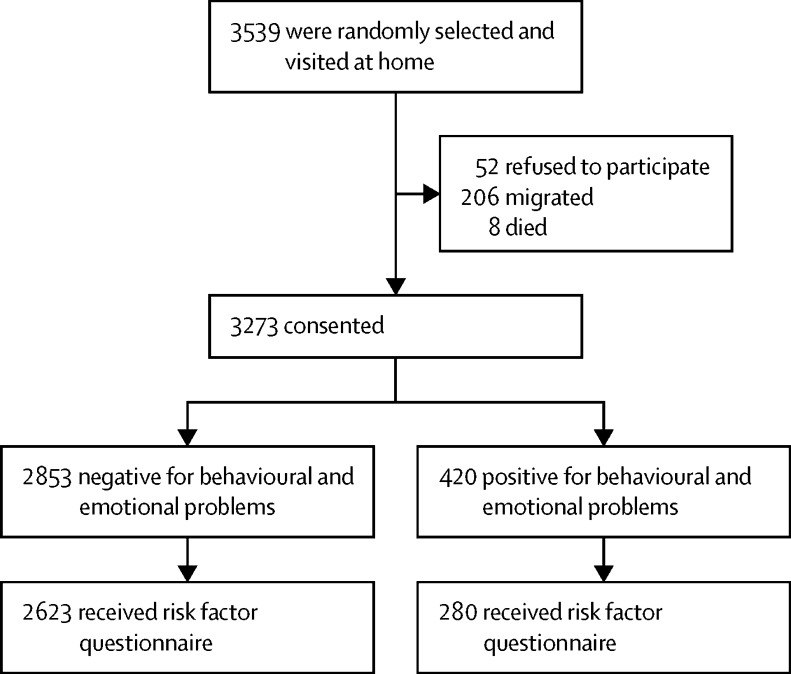
Derivation of the sample for the behavioural and emotional survey Fieldworkers visited 3539 children in their homes, of whom 3273 (93%) consented to take part in the epidemiological survey.

3539 parents or guardians of preschool children were randomly selected from KHDSS to take part in this study. Of these, 3273 (93%) families consented to participate in the behavioural survey and were visited at home and we administered the CBCL to the parents or guardians of children in these families (figure 1). Of the 3273 children in the study, 1671 (51%) were males. The median age was 4 years (IQR 3–5). The sex and median age did not differ by region of the KHDSS (table 1). However, statistically significant differences between the northern and southern regions of the KHDSS were observed for sociodemographic factors (eg, maternal religion), socioeconomic factors (eg, livestock owned), and medical history (eg, perinatal complications) among the 2903 (89%) children who received a risk factor questionnaire (table 1).

The mean total CBCL scores were 33·6 (SD 22·9), and the median scores were 27 (IQR 18–42) and did not differ between sexes (p=0·593). The median and mean CBCL scores for specific CBCL syndromes and DSM-oriented scales are shown in table 2. The distribution of raw CBCL scores was skewed, but log transformation appeared to normalise the scores (appendix).

The internal consistency of the 99 items of CBCL was excellent (α=0·95 [95% CI 0·94–0·96]), and remained unchanged when administered in Kiswahili (α=0·95 [0·94–0·95]) or Giriama (α=0·94 [0·93–0·95]). The internal consistency of externalising problems, internalising problems, the six CBCL syndromes, and the five DSM-oriented scales are shown in the appendix and are similar to those of an earlier pilot study from the same area.^13^

**Figure 2:**
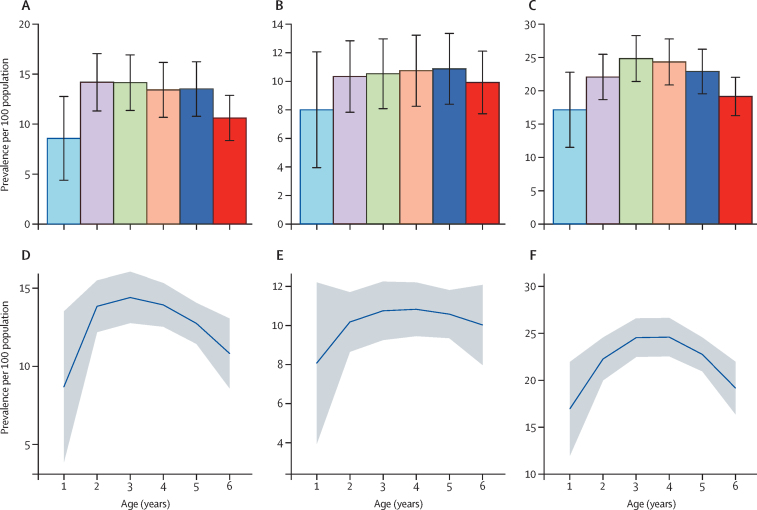
Prevalence of total CBCL problems, externalising problems, and internalising problems among 3273 preschool children living on the Kenyan coast The prevalence of total behavioural and emotional problems (A), externalising problems (B), and internalising problems (C) is provided by age group. Smoothened predictions of prevalence of behavioural and emotional problems (D), externalising problems (E), and internalising problems (F) were computed with a fractional polynomial equation. CBCL=Child Behaviour Checklist.

Total behavioural and emotional problems occurred in 420 (13%) children assessed with the CBCL—ie, a crude prevalence of 13% (95% CI 12–14). The prevalence did not differ by age (prevalence ratio [PR] 0·97, 95% CI 0·92–1·03; figure 2). The overall age-adjusted and sex-adjusted prevalence of behavioural and emotional problems was 11% (95% CI 10–13). Occurrence of these problems was similar in boys and girls (231 [14%] of 1671 in boys *vs* 189 [12%] of 1602 in girls, p=0·083).

Externalising problems occurred in 338 (10%) of 3273 children assessed with the CBCL, which is a crude prevalence of 10% (95% CI 9–11). The prevalence did not differ by age (PR 1·01, 95% CI 0·95–1·08; figure 2). The age and sex-adjusted prevalence of externalising problems was 9% (10–11). Externalising problems were significantly more frequent in boys than in girls (204 [12%] of 1671 in boys *vs* 134 [8%] of 1602 in girls, p<0·0001) but were similar in children 3 years or younger and in children older than 3 years (137 [10%] of 1353 *vs* 201 [11%] of 1920, p=0·751).

Internalising problems occurred in 728 (22%) children—ie, a crude prevalence of 22% (95% CI 21–24). The prevalence did not differ by age (PR 0·98, 95% CI 0·95–1·02; figure 2). The age-adjusted and sex-adjusted prevalence of internalising problems was 23% (95% CI 20–25). The occurrence of internalising problems was similar in boys and girls (367 [23%] of 1602 in boys *vs* 361 [22%] of 1671 in girls, p=0·369), and in children 3 years or younger and in children older than 3 years (306 [23%] of 1353 *vs* 422 [22%] of 1920, respectively, p=0·666).

The two most common CBCL syndromes were somatic problems (21%, 95% CI 20–23) and attention problems (16%, 14–17), while the two least frequent syndromes were aggressive behaviour (6%, 5–7) and sleep problems (4%, 3–4). The prevalences of other CBCL syndromes are shown in table 2. The commonest DSM-IV-oriented scale was anxiety problems (13%, 12–14) and the least common was oppositional defiant problems (2%, 2–3).

Factors associated with an increased risk of behavioural and emotional problems are listed by domain in table 3. The most important significant associations with the total score for behavioural and emotional problems among the pregnancy and birth factors were adverse perinatal events and abnormal pregnancy (table 3). Epilepsy and acute seizures were significantly associated with total CBCL problems among the seizure factors (table 3). Of the medical history factors, cassava consumption, head injury, and eating of soil were associated with total problems (table 3). The levels of house status, water availability, and toilet type were associated with total problems and the risk ratios for each level are shown in table 3. Externalising and internalising factors were associated with similar risk factors identified for total problems, but their effect size was smaller than those for total CBCL problems. (appendix).

There was an increased prevalence of clinically confirmed acute seizures (35 [14%] of 245 *vs* 85 [3%] of 2546, p<0·0001) and epilepsy (11 [5%] of 223 *vs* 11 [<1%] of 2476, p<0·0001) in those with total CBCL problems compared with those without. A few other comorbidities were significantly more frequent in those with behavioural and emotional problems compared with those without: burns for total problems and externalising problems, skin bruises and scars for total problems, and respiratory problems for internalising problems. The occurrence of other medical comorbidities is shown in table 4.

## Discussion

To our knowledge, this study is the largest epidemiological study of behavioural and emotional problems in young African children. Total behavioural and emotional problems as measured by the CBCL occurred in 13% of preschool children, with internalising problems more frequent than externalising problems (22% *vs* 10%). These problems were associated with multiple risk factors, and other medical problems such as seizures.

The prevalence of total problems (13%) is similar to that of preschool studies from other settings in low-and-middle-income countries such as Turkey (11·9%, assessed with the CBCL),^19^ and high-income countries such as Germany (12·4%, assessed with the CBCL).^20^ The prevalence remained high even after accounting for age and sex. The prevalence was higher than in Romania (8·8%, assessed with the CBCL),^21^ and Norway (7·1%, screened with the Strengths and Difficulties Questionnaire [SDQ]).^22^ The differences could be partly explained by the different assessment tools across studies or that symptoms considered behavioural and emotional problems in one culture may not be problematic in another.

Internalising problems were more prevalent than externalising problems (22% *vs* 10%), similar to preschool studies from New Zealand^23^ and Germany.^21^ These differences might reflect the fact that the CBCL has more questions and syndromes dedicated to internalising problems,^2^ which improves the sensitivity. This is a strength of the CBCL because internalising problems are less conspicuous and thus are more likely to be under-reported by parents.^24^

There was a heterogeneous prevalence of specific syndromes and DSM-oriented scales as found in other studies. Somatic problems were the most common CBCL syndrome, but this finding might be confounded by inclusion of other behavioural and emotional problems that parents cannot comprehend, or by febrile illnesses, which are common in this rural population. Many caregivers in this area are women with other multiple family responsibilities and think that children requiring increased care have attention and anxiety problems, which were common. The prevalence of ADHD problems was 5%, which is similar to estimates from a meta-analysis of studies across the world (5·3%).^25^ Most of these behavioural and emotional problems might be prevented by the initiation of parental interventions such as the Incredible Years Parent-Toddler programme or the Triple P-positive parenting programme.^7^, ^26^

Seizure disorders, including epilepsy and acute seizures, were important risk factors for behavioural and emotional problems. This association was established in previous studies of epilepsy,^27^ and the poor outcomes could be due to the seizures, underlying neurological impairment, and a shared genetic susceptibility or cause. Risk ratios for epilepsy were significantly greater than those for acute seizures, as would be expected since the former is a chronic condition with serious health and psychosocial consequences.

Only a few studies in high-income countries have found an association between behavioural and emotional problems and febrile seizures,^10^ but these studies were published nearly 20 years ago. Acute seizures in our study could be symptomatic of an underlying CNS cause such as malaria,^28^ which can increase the risk of developing behavioural and emotional outcomes. The role of seizure disorders in behavioural and emotional problems might be mediated or moderated by other non-seizure factors, implying that control of many risk factors would have maximum public health benefits.

The association of antenatal and perinatal problems with behavioural and emotional problems is mediated through seizures or underlying neurological impairment.^29^ The associations of consumption of cassava and soil with behavioural and emotional problems are probably mediated through social disadvantage and neurological impairments including seizures, although both factors might be directly neurotoxic,^30^ or are a consequence of the behavioural and emotional problems.

History of hospital admission with febrile illnesses and head injury were associated with behavioural and emotional problems, and might reflect that these problems are related to neurological insults, including those from infections such as falciparum malaria. Pets are intermediate hosts for parasitic infections such as *Toxoplasma gondii*, which are associated with mental health problems in humans.^31^ Poor house condition is an important marker of social disadvantage and inequalities in health-care access. Mothers who cannot afford antenatal services will deliver at home, increasing the risk of perinatal complications, which were associated with behavioural and emotional problems. Initiation of interventions aimed at socioeconomic empowerment could reduce mental health problems.

Comorbidities were common in behavioural and emotional problems, but only a few children showed significant differences when compared with those without these problems. Seizure disorders were particularly common in children with behavioural and emotional problems, occurring in about 20% of children, and their association with behavioural and emotional problems is well recognised in the literature.^27^ Burns were strikingly common in children with externalising problems who are often restless, increasing the risk of injuries including burns. Behavioural and emotional problems also co-occurred with respiratory problems and skin problems. Common comorbidities of behavioural and emotional problems should be assessed and managed in young children.

The strength of this study is based on a large randomly selected community sample that is adequately powered to estimate prevalence and identify underlying risk factors. The assessment was performed using the CBCL, which is an internationally recognised tool that was formally adapted and validated in the local population.

This is a cross-sectional study, in which the causal inference of associations is difficult without prospective longitudinal studies. We did not assess the family stressors, child maltreatment, and maternal mental health and HIV/AIDS status that might influence behavioural and emotional outcomes of children, although we adjusted for other maternal-related factors.

Overall, there is substantial burden of behavioural and emotional problems in young children living in rural Kenya and this should be addressed with appropriate interventions. Internalising problems were more common than externalising problems as in other studies that used the CBCL. These problems are as common as those in older children and in high-income countries.^5^ The burden of behavioural and emotional problems might be reduced by controlling risk factors, for example encouraging use of antenatal services to prevent mental health problems associated with perinatal complications. Common comorbidities of behavioural and emotional problems should be assessed and managed. There is a need to set up future studies to understand the role of genetics, family violence, child maltreatment, and maternal mental health and HIV/AIDS status in the risk of behavioural and emotional problems; determine risk of psychopathology later in life; and assess the effectiveness of parent-family interventions in addressing mental health problems.

## Figures and Tables

**Table 1:** Sociodemographic, socioeconomic, and medical characteristics of study participants by demographic surveillance area

			**Demographic surveillance region**		**Total (n=3273)**	p value*
			Northern (n=1437)	Southern (n=1836)		
Child's age (months)			47 (31–64)	48 (32–64)	48 (32–64)	0·264
Male sex			731 (51%)	934 (51%)	1671 (51%)	0·813
Mother's age (years)			29 (25–35)	30 (25–35)	30 (25–35)	0·156
Administered a risk factor questionnaire			1303 (90%)	1600 (87%)	2903 (89%)	..
Sociodemographic information						
	Mother's marital status					0·017
		Single	178 (14%)	188 (12%)	366 (13%)	..
		Separated or divorced	80 (6%)	73 (5%)	153 (5%)	..
		Widowed	8 (1%)	22 (1%)	30 (1%)	..
		Married	1037 (80%)	1317 (82%)	2354 (81%)	..
	Mother's religion					<0·0001
		None	318 (24%)	373 (23%)	691 (24%)	..
		Traditional	77 (6%)	111 (7)	188 (6%)	..
		Islam	116 (9%)	227 (14%)	343 (12%)	..
		Christian	792 (61%)	889 (56%)	1681 (58%)	..
	Mother's education level					0·720
		None	447 (34%)	558 (35%)	1005 (35%)	..
		Primary	753 (58%)	930 (58%)	1683 (58%)	..
		Secondary	83 (6%)	86 (5%)	169 (6%)	..
		Tertiary	20 (2%)	26 (2%)	46 (2%)	..
	Mother's ethnicity					<0·0001
		Mijikenda	59 (5%)	97 (6%)	156 (5%)	..
		Other Mijikenda	55 (4%)	120 (8%)	175 (6%)	..
		Kauma	72 (6%)	198 (12%)	270 (9%)	..
		Chonyi	183 (14%)	693 (43%)	876 (30%)	..
		Giriama	934 (72%)	492 (31%)	1426 (49%)	..
	Economic activity (mother)		955 (73%)	1159 (72%)	2114 (73%)	0·607
	Economic activity (father)		1111/1196 (93%)	1371/1488 (92%)	2482/2684 (92%)	0·461
	Number of children		4 (3–6)	4 (3–6)	4 (3–6)	0·841
Socioeconomic status and household information						
	Water availability					<0·0001
		Infrequent	328 (25%)	487 (30%)	815 (28%)	..
		Weekly	153 (12%)	48 (3%)	201 (7%)	..
		Daily	328 (25%)	364 (23%)	692 (24%)	..
		Always	494 (38%)	701 (44%)	1195 (41%)	..
	House status					..
		Dilapidated	130 (10%)	178 (11%)	308 (11%)	0·053
		Major repairs needed	90 (8%)	84 (5%)	183 (6%)	..
		Incompletely built	24 (2%)	25 (2%)	49 (2%)	..
		Minor or no repairs needed	1050 (81%)	1313 (82%)	2363 (81%)	..
	Toilet type					<0·0001
		Bush or none	522 (40%)	479 (30%)	1001 (34%)	..
		Traditional pit	624 (48%)	913 (57%)	1537 (53%)	..
		Ventilated pit	86 (7%)	133 (8%)	219 (8%)	..
		Flush	71 (5%)	75 (5%)	146 (5%)	..
	Livestock owned					<0·0001
		None	516 (40%)	841 (53%)	1357 (47%)	..
		<5	324 (25%)	265 (17%)	589 (20%)	..
		>5	463 (36%)	494 (31%)	957 (33%)	..
Pregnancy and birth history						
	Pregnancy problems		112 (9%)	78 (5%)	190 (7%)	<0·0001
	Mother's age at first birth		18 (17–20)	18 (17–20)	18 (17–20)	0·334
	Delivery at home		833 (64%)	923 (58%)	1756 (60%)	0·001
	Adverse perinatal events		37 (3%)	28 (2%)	65 (2%)	0·048
Medical history						
	Family history of seizures		127 (10%)	125 (8%)	252 (9%)	0·066
	Family history of febrile seizures		95 (7%)	147 (9%)	242 (8%)	0·066
	Previous hospital admission		124 (10%)	146 (9%)	270 (9%)	0·72
	Head injury		24 (2%)	24 (2%)	48 (2%)	0·47
	Dogs or cats in compound		541 (42%)	697 (44%)	1238 (43%)	0·27
	Eats cassava		990 (76%)	1179 (74%)	2169 (75%)	0·16
	Eats soil		175 (13%)	153 (10%)	328 (11%)	0·001
	Eats pork		134 (10%)	134 (8%)	268 (9%)	0·077
	Bednet use		1116 (86%)	1329 (83%)	2445 (84%)	0·057
	Snores at night		259 (20%)	319 (20%)	578 (20%)	0·97
	Deceased father		35/1110 (3%)	43/1362 (3%)	78/2472 (3%)	0·97

Data are median (IQR), n (%), or n/N (%). CBCL=Child Behaviour Checklist.

* Comparison of characteristics between the northern demographic surveillance area and southern demographic surveillance area. The questionnaire for sociodemographic characteristics, socioeconomic status, and medical history was administered to 2903 (89%) of the 3273 individuals screened with CBCL.

**Table 2:** Behavioural and emotional scores and problems for the CBCL seven-syndrome scales and the DSM-IV-oriented scales

	**Median score (IQR)**	**Mean scores (SD)**	**Number of children with problems (n=3273); n (%; 95% CI)**
**CBCL syndromes**			
Emotionally reactive	2 (1–3)	2·4 (2·4)	331 (10·1%; 9·1–11·2)
Anxious or depressed	2 (0–4)	2·8 (3·1)	417 (12·7%; 11·6–13·9)
Somatic problems	2 (1–3)	3·1 (2·6)	695 (21·2%; 19·8–22·7)
Withdrawn	2 (0–3)	2·1 (2·0)	360 (11·0%; 9·9–12·1)
Sleep problems	2 (1–3)	2·3 (2·2)	114 (3·5%; 2·9–4·2)
Attention problems	3 (2–5)	3·4 (2·0)	507 (15·4%; 14·3–16·8)
Aggressive behaviour	6 (3–10)	7·5 (6·6)	189 (5·8%; 5·0–6·6)
**DSM-IV-oriented**			
Affective problems	1 (0–3)	1·9 (2·5)	189 (5·8%; 5·0–6·6)
Anxiety problems	4 (2–6)	4·5 (3·5)	413 (12·6%; 11·5–13·8)
Pervasive developmental problems	2 (1–4)	3·1 (2·9)	173 (5·3%; 4·5–6·1)
Attention-deficit hyperactivity disorder problems	5 (3–7)	5·5 (2·9)	163 (5·0%; 4·3–5·8)
Oppositional problems	2 (0–3)	2·2 (2·3)	76 (2·3%; 1·8–2·9)

CBCL=Child Behaviour Checklist. DSM-IV=Diagnostic and Statistical Manual of Mental Disorders, 4th edn.

**Table 3:** Factors associated with total behavioural and emotional problems in 2903 children who received the risk factor questionnaire

		**Frequency distribution**		Penultimate adjusted model*	**Final adjusted model**†	Penultimate adjusted model*	**Final adjusted model**†
		Number of children with no problems (n=2623)	Number of children with problems (n=280)	Risk ratio (95% CI)	Risk ratio (95% CI)	β coefficient (95% CI)	β coefficient (95% CI)
**Pregnancy and birth information**							
Mother's age at first birth (years)		18 (17–20)	18 (16–20)	0·98 (0·96 to 1·01)	1·01 (0·98 to 1·06)	–0·01 (–0·01 to 0·00)	0·00 (–0·01 to 0·01)
Pregnancy problems		155 (6%)	35 (13%)	2·03 (1·46 to 2·81)	2·11 (1·55 to 2·88)	0·25 (0·15 to 0·36)	0·27 (0·17 to 0·38)
Delivery at home		1583 (60%)	173 (62%)	1·07 (0·85 to 1·34)	1·00 (0·78 to 1·29)	0·00 (–0·05 to 0·04)	–1·57 (–3·34 to 0·20)
Adverse perinatal events		34 (1%)	31 (11%)	5·41 (4·07 to 7·20)	4·34 (3·21 to 5·87)	0·69 (0·55 to 0·84)	0·67 (0·53 to 0·82)
**Seizure information**							
Any seizure disorder		157 (6%)	68 (24%)	3·83 (3·02 to 4·85)	2·90 (2·24 to 3·77)	0·42 (0·33 to 0·51)	0·39 (0·30 to 0·49)
Acute seizures		85/2546 (3%)	35/245 (14%)	3·72 (2·74 to 5·08)	2·62 (1·89 to 3·64)	0·36 (0·24 to 0·49)	0·33 (0·21 to 0·46)
Epilepsy		11/2476 (<1%)	11/223 (5%)	6·31 (4·11 to 9·70)	4·71 (2·65 to 8·35)	0·68 (0·38 to 0·99)	0·60 (0·28–0·93)
Family history of seizures		204 (8%)	48 (17%)	2·19 (1·65 to 2·91)	2·00 (1·46 to 2·62)	0·18 (0·10 to 0·27)	0·21 (0·11 to 0·30)
Family history of febrile seizures		146 (6%)	96 (34%)	5·73 (4·65 to 7·07)	4·07 (3·21 to 5·18)	0·63 (0·55 to 0·71)	0·61 (0·52 to 0·70)
**Medical history information**							
Previous hospital admission		211 (8%)	59 (21%)	2·63 (2·03 to 3·41)	2·25 (1·72–2·95)	0·25 (0·17 to 0·33)	0·24 (0·15 to 0·34)
Head injury		26 (1%)	22 (8%)	5·14 (3·68 to 7·19)	3·11 (2·19 to 4·43)	0·64 (0·47 to 0·81)	0·56 (0·38 to 0·74)
Eats cassava		1905 (73%)	264 (94%)	5·58 (3·40 to 9·18)	5·68 (3·22 to 10·03)	0·30 (0·26 to 0·35)	0·33 (0·28 to 0·39)
Dogs or cats in compound		1103 (42%)	135 (48%)	1·25 (0·99 to 1·56)	1·30 (1·04 to 1·64)	0·06 (0·02 to 0·11)	0·06 (0·01 to 0·12)
Eats soil		251 (10%)	77 (28%)	3·14 (2·44 to 4·03)	2·80 (2·16 to 3·64)	0·42 (0·35 to 0·50)	0·40 (0·32 to 0·48)
Snores at night		462 (18%)	116 (41%)	2·83 (2·28 to 3·53)	2·23 (1·77 to 2·81)	0·32 (0·26 to 0·38)	0·29 (0·23 to 0·37)
Eats pork		228 (9%)	40 (14%)	1·65 (1·21 to 2·52)	1·74 (1·28 to 2·37)	0·13 (0·04 to 0·22)	0·06 (0·02 to 0·11)
Bednet use		2198 (84%)	247 (88%)	1·38 (0·97 to 1·97)	1·35 (0·91 to 1·99)	0·07 (0·01 to 0·14)	0·02 (–0·05 to 0·09)
Malnutrition		13/377 (6%)	6/243 (2%)	0·72 (0·36 to 1·52)	1·01 (0·47 to 2·16)	–0·18 (–0·53 to 0·16)	–0·05 (–0·42 to 0·33)
**Socioeconomic information**							
Water availability							
	Infrequent	662 (25%)	153 (55%)	1·00	1·00‡	0·00	0·00‡
	Weekly	167 (6%)	34 (12%)	0·90 (0·63 to 1·29)	1·41 (0·99 to 2·00)	−0·25 (−0·36 to −0·13)	−0·13 (−0·24 to −0·02)
	Daily	670 (26%)	22 (8%)	0·17 (0·11 to 0·26)	0·25 (0·15 to 0·41)	−0·38 (−0·44 to −0·32)	−0·36 (−0·43 to −0·29)
	Always	1124 (43%)	71 (25%)	0·31 (0·24 to 0·41)	0·42 (0·31 to 0·56)	−0·27 (−0·32 to −0·22)	−0·23 (−0·29 to −0·18)
House status							
	Dilapidated	159 (6%)	149 (53%)	1·00‡	1·00‡	0·00‡	0·00‡
	Major repairs needed	149 (6%)	34 (12%)	0·38 (0·28 to 0·53)	0·51 (0·35 to 0·74)	−0·62 (−0·75 to −0·50)	−0·58 (−0·73 to −0·43)
	Incompletely built	45 (2%)	4 (1%)	0·17 (0·07 to 0·43)	0·18 (0·06 to 0·54)	−0·77 (−0·97 to −0·57)	−0·84 (−10·5 to −0·62)
	Minor or no repairs needed	2270 (87%)	93 (33%)	0·08 (0·06 to 0·10)	0·11 (0·08 to 0·14)	−0·84 (−0·91 to −0·78)	−0·85 (−0·93 to −0·78)
Toilet type							
	Bush or none	816 (31%)	185 (66%)	1·00‡	1·00‡	0·00‡	0·00‡
	Traditional pit	1466 (56%)	71 (25%)	0·24 (0·19 to 0·32)	0·31 (0·23 to 0·41)	−0·26 (−0·31 to −0·21)	−0·27 (−0·32 to −0·21)
	Ventilated pit	202 (8%)	17 (6%)	0·41 (0·25 to 0·65)	0·55 (0·33 to 0·93)	−0·28 (−0·38 to −0·18)	−0·30 (−0·40 to −0·19)
	Flush	139 (5%)	7 (3%)	0·25 (0·12 to 0·53)	0·46 (0·20 to 1·04)	−0·18 (−0·28 to −0·09)	−0·16 (−0·27 to −0·05)
Livestock owned‡							
	None	1214 (46%)	143 (51%)	1·00‡	1·00‡	0·00‡	0·00‡
	<5	522 (20%)	67 (24%)	1·07 (0·82 to 1·42)	0·95 (0·70 to 1·27)	−0·04 (−0·10 to 0·03)	0·05 (−0·11 to 0·03)
	>5	887 (34%)	70 (25%)	0·69 (0·52 to 0·91)	0·66 (0·50 to 0·89)	−0·07 (−0·12 to −0·02)	−0·05 (−0·11 to −0·00)
Number of siblings		4 (3–6)	4 (3–6)	1·01 (0·97 to 1·05)	0·95 (0·88 to 1·01)	0·00 (−0·01 to 0·01)	−0·02 (0·03 to 0·00)
Deceased father		73 (3%)	5 (4%)	1·15 (0·48 to 2·73)	1·49 (0·61 to 3·61)	−0·09 (−0·22 to 0·04)	0·04 (−0·15 to 0·23)

Data are median (IQR), n (95% CI), n (%), n/N (%).

* Adjusted for child factors (age, sex, schooling, and region of residence).

† Adjusted for both child factors (age, sex, schooling, region of residence) and maternal factors (age, marital status, education level, economic or employment status, religion, and ethnicity).

‡ The test for linear trend for the levels of the factor was statistically significant. Associations for categorical variables (eg, behavioural and emotional problems) were reported as risk ratios, whereas for continuous scores regression coefficients were used.

**Table 4:** Medical and neurocognitive comorbidities of behavioural and emotional problems by age group in children randomly selected for clinical assessment

	**All children (n=620)**	**Total problems**			**Externalising problems**			**Internalising problems**		
		No (n=377)	Yes (n=243)	p value	No (n=424)	Yes (n=196)	p value	No (n=266)	Yes (n=354)	p value
Hypoaesthesia*	29 (5%)	13 (3%)	16 (6%)	0·071	17 (4%)	12 (6%)	0·247	8 (3%)	21 (6%)	0·088
Abnormal limb function†	8 (1%)	3 (1%)	5 (2%)	0·174	4 (1%)	4 (2%)	0·260	2 (1%)	6 (2%)	0·303
Motor impairments‡	86 (14%)	46 (12%)	40 (16%)	0·134	56 (13%)	30 (15%)	0·482	36 (14%)	50 (15%)	0·833
Intellectual disability	27 (4%)	12 (3%)	15 (6%)	0·075	15 (4%)	12 (6%)	0·143	8 (3%)	19 (5%)	0·154
Otitis and ear canal abnormalities	26 (4%)	18 (5%)	8 (3%)	0·418	22 (5%)	4 (2%)	0·084	12 (5%)	14 (4%)	0·840
Cardiovascular problems	4 (1%)	3 (1%)	1 (<1%)	1·000	4 (1%)	0 (0%)	0·313	1 (<1%)	3 (1%)	0·639
Respiratory problems	11 (2%)	6 (2%)	5 (2%)	0·759	6 (1%)	5 (3%)	0·337	1 (<1%)	10 (3%)	0·023
Malnutrition	19 (3%)	13 (3%)	6 (2%)	0·490	16 (4%)	3 (2%)	0·132	9 (4%)	10 (3%)	0·690
Skin scars or bruises	20 (3%)	8 (2%)	12 (5%)	0·053	11 (3%)	9 (5%)	0·191	7 (4%)	13 (4%)	0·468
Burn marks	65 (10%)	32 (8%)	33 (14%)	0·043	32 (8%)	33 (17%)	<0·0001	26 (10%)	39 (11%)	0·619

* Reduced sensation to touch or painful stimuli.

† Reduced or increased reflex activity.

‡ Both general and fine motor skills assessed.
