# Supplementary material for: Burden, risk factors, and comorbidities of behavioural and emotional problems in Kenyan children: a population-based study
Source: Lancet Psychiatry. 2017 Feb;4(2):136–45. doi: 10.1016/S2215-0366(16)30403-5 (PMC5285446; doi:10.1016/S2215-0366(16)30403-5)

THE LANCET
Psychiatry

Supplementary appendix

This appendix formed part of the original submission and has been peer reviewed. We post it as supplied by the authors.

Supplement to: Kariuki S M, Abubakar A, Kombe M, et al. Burden, risk factors, and comorbidities of behavioural and emotional problems in Kenyan children: a population-based study. *Lancet Psychiatry* 2017; **4:** 136–45.

Supplementary Materials

Supplementary table 1. Criteria for constructing CBCL syndromes and DSM-IV-oriented scales using the 99 items ADH=attention-deficit hyperactivity disorder

CBCL syndromes							DSM-oriented scales				
**Emotionally-reactive**	**Anxious-depressed**	**Somatic complaints**	**Withdrawn**	**Sleep problems**	**Attention problems**	**Aggressive behaviour**	**Affective problems**	**Anxiety problems**	**Pervasive developmental problems**	**ADH problems**	**Oppositional defiant problems**
Disturbed by change	Clings	Aches	Acts too young	Doesn't want to sleep alone	Can't concentrate	Can't stand waiting	Cries	Clings	Afraid to try new	Can't concentrate	Defiant
Twitches	Feelings hurt	Can't stand things out of place	Avoids eye contact	Trouble sleeping	Can't sit still	Defiant	Doesn't eat well	Doesn't want to sleep alone	Avoids eye contact	Can't sit still	Disobedient
Panics	Upset by separation	Constipated	Doesn't answer	Nightmares	Clumsy	Demands met	Trouble sleeping	Doesn't leave home	Can't stand things out of place	Can't stand waiting	Angry moods
Swifts between sad-excite	Looks unhappy	Diarrhoea	Refuses active games	Resists bed	Quickly shifts	Destroys other's	Looks unhappy	Fears	Disturbed by change	Demands must be met	Stubborn
Moody	Nervous	Doesn't eat well	Unresponsive to affection	Sleeps little	Wanders away	Disobedient	Overeating	Upset by separation	Doesn't answer	Gets into everything	Temper
Sulks	Self-conscious	Headaches	Little affection	Talks, cries in sleep		Lacks guilt	Overtired	Nervous	Doesn't get along with peers	Quickly shifts	Uncooperative
Upset by new	Fearful	Nausea	Little interest	Wakes often		Easily frustrated	Little interest	Nightmares	Rocks head, body		
Whining	Sad	Painful bowel	Withdrawn			Fights	Sleeps little	Panics	Unresponsive to affection		
Worries		Stomach aches				Hits others	Underactiv e	Fearful	Little affection		
		Too concerned with neat/clean				Hurts accidentally	Sad	Worries	Speech problem		
		Vomits				Angry moods			Strange behaviour		
						Attacks people			Upset by new		
						Punishment doesn't change			Withdrawn		
						Screams					
						Selfish					
						Stubborn					
						Temper					
						Uncooperative					
						Wants attention					

Supplementary table 2. Internal consistency Cronbach's alpha for the CBCL total scale, externalising scale, internalising scale and seven-syndromes scale by local language

CBCL scale	Kiswahili (N=3,037)	Giriama (N=236)	All children (N=3,273)
**Total scale**	0.95 (0.94-0.96)	0.94 (0.93-0.95)	0.95 (0.94-0.96)
Externalising scale (95%CI)	0.86 (0.84-0.86)	0.86 (0.83-0.87)	0.86 (0.84-0.88)
Internalising scale (95%CI)	0.89 (0.87-0.91)	0.86 (0.84-0.88)	0.88 (0.87-0.89)
**CBCL syndrome scales**			
Emotionally-reactive (95%CI)	0.68 (0.67-0.69)	0.60 (0.53-0.67)	0.68 (0.66-0.70)
Anxious-depressed (95%CI)	0.76 (0.75-0.77)	0.76 (0.72-0.80)	0.76 (0.74-0.76)
Somatic problems (95%CI)	0.69 (0.67-0.71)	0.67 (0.61-0.73)	0.68 (0.66-0.70)
Withdrawn (95%CI)	0.55 (0.53-0.57)	0.55 (0.47-0.63)	0.55 (0.52-0.58)
Sleep problems (95%CI)	0.65 (0.64-0.66)	0.71 (0.65-0.77)	0.66 (0.64-0.68)
Attention problems (95%CI)	0.50 (0.48-0.52)	0.58 (0.51-0.65)	0.50 (0.48-0.52)
Aggressive behaviour (95%CI)	0.85 (0.84-0.86)	0.85 (0.83-0.87)	0.85 (0.84-0.86)
**DSM-Oriented scales**			
Affective problems	0.67 (0.64-0.70)	0.65 (0.59-0.71)	0.67 (0.66-0.68)
Anxiety problems	0.73 (0.71-)	0.69 (0.65-0.73)	0.73 (0.71-0.75)
Pervasive developmental problems	0.60 (0.58-0.62)	0.62 (0.56-0.68)	0.63 (0.61-0.64)
Attention deficit/hyperactivity problems	0.62 (0.60-0.64)	0.62 (0.55-0.69)	0.63 (0.60-0.66)
Oppositional defiant problems	0.65 (0.63-0.67)	0.73 (0.68-0.78)	0.66 (0.64-0.68)

CI=confidence interval

Supplementary table 3. Factors associated with externalising problems among 2,903 children who received the risk factor questionnaire

	Frequency distribution		Penultimate adjusted model[a]		Final adjusted model[b]	
**Factor**	**No externalising problem (N=2666)**	**Externalising problems (N=237)**	**Risk ratio (95%CI), p-value**	**β Coefficient (95%CI), p-value**	**Risk ratio (95%CI)**	**β Coefficient (95%CI)**
**Pregnancy and birth information**						
Primigravida age	18 (17-20)	18 (16-20)	**0.97 (0.94-1.00)**	-0.01 (-0.02, 0.00)	1.02 (0.97-1.07)	-0.01 (-0.02, 0.00)
Abnormal pregnancy	162 (6%)	28 (12%)	**1.91 (1.32-2.76)**	**0.17 (0.05, 0.28)**	**2.02 (1.42-2.87)**	**0.18 (0.06, 0.31)**
Delivery at home	1616 (61%)	140 (59%)	0.95 (0.74-1.21)	0.03 (-0.02, 0.09)	**0.75 (0.57-0.97)**	0.01 (-0.05, 0.07)
Adverse perinatal events	43 (1%)	22 (9%)	**4.41 (3.05-6.39)**	**0.60 (0.42, 0.79)**	**3.45 (2.42-4.92)**	**0.55 (0.35, 0.74)**
**Seizures information**						
Any seizure disorder	175 (7%)	50 (21%)	**3.71 (2.40-4.20)**	**0.39 (0.29, 0.50)**	**2.37 (1.76-3.19)**	**0.38 (0.27-0.49)**
Acute seizures	91/2577 (3%)	29/214 (14%)	**3.47 (2.46-4.91)**	**0.40 (0.26, 0.54)**	**2.48 (1.73-3.56)**	**0.37 (0.23, 0.52)**
Epilepsy	11/2504 (1%)	8/195 (4%)	**5.23 (2.96-9.23)**	**0.64 (0.36, 0.92)**	**3.80 (1.98-7.30)**	**0.55 (0.25-0.84)**
Family history of seizures	209 (8%)	43 (17%)	**2.35 (1.74-3.19)**	**0.17 (0.06, 0.27)**	**2.18 (1.60-2.96)**	**0.19 (0.07, 0.30)**
Family history of febrile seizures	166 (6%)	76 (32%)	**5.19 (4.09-6.59)**	**0.62 (0.53, 0.71)**	**3.83 (2.89-5.08)**	**0.58 (0.48, 0.69)**
**Medical history information**						
Previous hospitalisation	224 (8%)	46 (19%)	**2.32 (1.72-3.13)**	**0.19 (0.09, 0.29)**	**1.94 (1.42-2.66)**	**0.18 (0.07-0.29)**
Head injury	30 (1%)	18 (8%)	**4.73 (3.16-7.08)**	**0.67 (0.47, 0.86)**	**2.61 (1.63-4.21)**	**0.54 (0.33, 0.75)**
Eats cassava	1946 (73%)	223 (94%)	**5.38 (3.15-9.16)**	**0.33 (0.27, 0.39)**	**4.67 (2.63-8.27)**	**0.18 (0.07, 0.29)**
Dogs/cats in compound	1124 (42%)	114 (48%)	1.25 (0.98-1.60)	0.05 (-0.01, 0.10)	1.26 (0.98-1.63)	0.04 (-0.02, 0.10)
Eats soil	270 (10%)	58 (24%)	**2.75 (2.06-3.66)**	**0.32 (0.23, 0.40)**	**2.32 (1.71-3.13)**	**0.29 (0.19, 0.38)**
Snores at night	475 (18%)	103 (43%)	**3.06 (2.41-3.90)**	**0.29 (0.22, 0.36)**	**2.32 (1.79-3.00)**	**0.25 (0.17, 0.33)**
Eats pork	233 (9%)	35 (15%)	**1.69 (1.21-2.37)**	0.04 (-0.06, 0.15)	**1.91 (1.37-2.66)**	0.04 (-0.07-0.16)
Bednet use	2235 (84%)	210 (89%)	1.45 (0.98-2.13)	**0.07 (0.01, 0.14)**	1.44 (0.93-2.22)	-0.02 (-0.10, 0.06)
**Socioeconomic information**						
**Water availability**						
Infrequent	684 (26%)	131 (55%)	**1.00[c]**	**0.00[c]**	**1.00[c]**	**0.00[c]**
Weekly	172 (6%)	29 (12%)	0.89 (0.60-1.31)	-0.08 (-0.19, 0.03)	1.44 (0.98-2.13)	-0.01 (-0.13, -0.10)
Daily	672 (25%)	20 (8%)	**0.18 (0.11-0.28)**	**-0.56 (-0.63, -0.49)**	**0.26 (0.15-0.43)**	**-0.56 (-0.64, -0.48)**
Always	1138 (43%)	57 (24%)	**0.29 (0.22-0.40)**	**-0.42 (-0.48, -0.36)**	**0.39 (0.28-0.54)**	**-0.40 (-0.46, -0.33)**
**House status**						
Dilapidated	182 (7%)	126 (53%)	**1.00[c]**	**0.00[c]**	1.00[c]	**0.00[c]**
Needs major repair	152 (6%)	31 (13%)	**0.41 (0.29-0.58)**	**-0.63 (-0.77, -0.48)**	**0.51 (0.33-0.79)**	**-0.57 (-0.74, -0.41)**
Under construction	45 (2%)	4 (1%)	**0.21 (0.08-0.58)**	**-0.75 (-1.00, -0.51)**	**0.23 (0.08-0.68)**	**-0.83 (-1.10, -0.56)**

Needs Minor repair	2287 (86%)	76 (32%)	**0.08 (0.06-0.10)**	**-0.84 (-0.91, -0.76)**	**0.11 (0.08-0.15)**	**-0.84 (-0.92, -0.76)**
**Toilet type**						
Bush/none	845 (32%)	156 (66%)	1.00[c]	0.00[c]	1.00[c]	0.00[c]
Traditional pit	1471 (55%)	66 (28%)	**0.27 (0.20-0.36)**	**-0.29 (-0.35, -0.23)**	**0.35 (0.27-0.48)**	**-0.30 (-0.37, -0.24)**
Ventilated pit	207 (8%)	12 (5%)	**0.34 (0.19-0.61)**	**-0.45 (-0.58, -0.34)**	**0.45 (0.23-0.86)**	**-0.53 (-0.67, -0.39)**
Flush	143 (5%)	3 (1%)	**0.13 (0.04-0.40)**	**-0.26 (-0.39, -0.14)**	**0.30 (0.09-0.93)**	**-0.29 (-0.44, -0.14)**
**Livestock owned[c]**						
None	1232 (46%)	125 (53%)	1.00[c]	0.00	1.00[c]	0.00[c]
<5	532 (20%)	57 (24%)	1.05 (0.77-1.42)	0.00 (-0.08, 0.08)	1.02 (0.75-1.40)	-0.01 (-0.10, 0.07)
>5	902 (34%)	55 (23%)	**0.62 (0.46-0.84)**	-0.05 (-0.10, 0.02)	**0.66 (0.50-0.89)**	-0.04 (-0.11, 0.02)
No of siblings	4 (3-6)	4 (3-6)	1.00 (0.95-1.05)	0.00 (-0.01, 0.02)	0.92 (0.85-1.00)	-0.01 (-0.03, 0.01)
Deceased father	73 (3%)	5 (4%)	1.31 (0.55-3.13)	-0.11 (-0.30, 0.08)	1.66 (0.70-3.93)	-0.01 (-0.03, 0.01)

Significant associations are highlighted in bold. [a]Adjusted for child factors (age, sex, schooling and region of residence). [b]Adjusted for both child factors (age, sex, schooling, region of residence) and maternal factors (age, marital status, education level, economic/employment status, religion and ethnicity). [c]The test for linear trend for the levels of the factor was statistically significant. Associations for categorical variables (e.g. behavioural and emotional problems) are reported as risk ratios, whereas for continuous scores regression coefficients are used.

Supplementary table 4. Factors associated with internalising problems among 2,903 children who received the risk factor questionnaire

	Frequency distribution		Penultimate adjusted model[a]		Final adjusted model[b]	
**Factor**	**No internalising problem (N=2368)**	**Internalising problems (N=535)**	**Risk ratio (95%CI), p-value**	**β Coefficient (95%CI), p-value**	**Risk ratio (95%CI)**	**β Coefficient (95%CI)**
**Pregnancy and birth information**						
Primigravida age	18 (17-20)	18 (16-20)	0.99 (0.97-1.01)	-0.01 (-0.02, 0.00)	1.02 (0.99-1.05)	**0.02 (0.00, 0.03)**
Abnormal pregnancy	117 (5%)	73 (14%)	**2.22 (1.82-2.72)**	**0.42 (0.30, 0.53)**	**2.25 (1.84-2.76)**	**0.46 (0.34, 0.58)**
Delivery at home	1423 (60%)	333 (62%)	1.06 (0.91-1.25)	-0.05 (-0.11, 0.01)	0.86 (0.73-1.03)	**-0.11 (-0.18, -0.04)**
Adverse perinatal events	28 (1%)	37 (7%)	**3.22 (2.56-4.03)**	**0.76 (0.58, 0.94)**	**2.94 (2.36-3.65)**	**0.79 (0.61, 0.97)**
**Seizures information**						
Any seizure disorder	128 (5%)	97 (18%)	**2.68 (2.25-3.19)**	**0.51 (0.40, 0.63)**	**2.27 (1.88-2.74)**	**0.51 (0.39-0.64)**
Acute seizures	70/2305 (3%)	50/486 (14%)	**2.61 (2.07-3.30)**	**0.41 (0.26, 0.56)**	**2.08 (1.62-2.68)**	**0.39 (0.22, 0.56)**
Epilepsy	7/2246 (1%)	15/453 (3%)	**4.15 (3.08-5.61)**	**0.80 (0.43, 1.17)**	**3.47 (2.42-4.99)**	**0.76 (0.37-1.15)**
Family history of seizures	180 (8%)	72 (13%)	**1.62 (1.31-2.01)**	**0.21 (0.10, 0.31)**	**1.60 (1.28-1.99)**	**0.23 (0.11, 0.36)**
Family history of febrile seizures	106 (4%)	136 (25%)	**3.79 (4.09-6.59)**	**0.73 (0.63, 0.83)**	**3.11 (2.62-3.67)**	**0.75 (0.63, 0.86)**
**Medical history information**						
Previous hospitalisation	184 (8%)	86 (16%)	**1.90 (1.56-2.31)**	**0.35 (0.25, 0.45)**	**1.76 (1.44-2.16)**	**0.39 (0.28-0.50)**
Head injury	18 (1%)	30 (6%)	**3.76 (2.99-4.73)**	**0.72 (0.54, 0.89)**	**2.80 (2.18-3.59)**	**0.68 (0.49, 0.87)**
Eats cassava	1702 (72%)	467 (87%)	**2.32 (1.82-2.95)**	**0.21 (0.15, 0.27)**	**2.44 (1.85-3.21)**	**0.20 (0.13, 0.27)**
Dogs/cats in compound	978 (41%)	260 (49%)	**1.27 (1.09-1.47)**	**0.13 (0.08, 0.19)**	**1.31 (1.11-1.54)**	**0.14 (0.07, 0.20)**
Eats soil	199 (8%)	129 (24%)	**2.48 (2.10-2.94)**	**0.47 (0.37, 0.56)**	**2.33 (1.96-2.78)**	**0.48 (0.37, 0.59)**
Snores at night	388 (16%)	190 (36%)	**2.22 (1.90-2.58)**	**0.34 (0.27, 0.42)**	**1.93 (1.64-2.26)**	**0.34 (0.26, 0.42)**
Eats pork	197 (8%)	71 (13%)	**1.51 (1.22-1.88)**	**0.28 (0.19, 0.38)**	**1.56 (1.24-1.95)**	0.33 (0.22-0.43)
Bednet use	1979 (84%)	466 (87%)	1.26 (0.99-1.58)	**0.09 (0.02, 0.18)**	1.08 (0.84-1.38)	0.10 (0.00, 0.10)
**Socioeconomic information**						
**Water availability**						
Infrequent	592 (25%)	233 (42%)	1.00[c]	0.00[c]	1.00[c]	0.00
Weekly	155 (7%)	46 (9%)	0.82 (0.61-1.09)	-0.14 (-0.28, 0.00)	1.17 (0.88-1.55)	-0.04 (-0.18, 0.11)
Daily	615 (26%)	77 (14%)	**0.40 (0.31-0.51)**	**-0.09 (-0.17, -0.01)**	**0.54 (0.41-0.70)**	-0.03 (-0.12, 0.06)
Always	1006 (42%)	189 (35%)	**0.57 (0.48-0.67)**	**-0.08 (-0.16, -0.01)**	**0.72 (0.60-0.86)**	-0.04 (-0.13, 0.04)
**House status**						
Dilapidated	96 (4%)	212 (40%)	1.00[c]	0.00[c]	1.00[c]	0.00[c]
Needs major repair	130 (5%)	53 (10%)	**0.41 (0.32-0.52)**	**-0.59 (-0.73, -0.44)**	**0.51 (0.39-0.66)**	**-0.59 (-0.76, -0.41)**
Under construction	41 (2%)	8 (2%)	**0.23 (0.12-0.43)**	**-0.80 (-1.04, -0.56)**	**0.24 (0.13-0.51)**	**-0.87 (-1.12, -0.61)**
Needs Minor repair	2101 (89%)	262 (49%)	**0.16 (0.14-0.18)**	**-0.94**	**0.18 (0.15-0.23)**	**-1.00 (-1.11, -0.90)**

				**(-1.03, -0.84)**		
**Toilet type**						
Bush/none	699 (30%)	302 (56%)	1.00[c]	0.00[c]	1.00[c]	0.00[c]
Traditional pit	1357 (57%)	180 (34%)	**0.39 (0.33-0.46)**	**-0.28 (-0.35, -0.21)**	**0.43 (0.36-0.52)**	**-0.28 (-0.36, -0.20)**
Ventilated pit	185 (8%)	34 (6%)	**0.51 (0.37-0.71)**	**-0.12 (-0.22, -0.11)**	**0.64 (0.45-0.90)**	**-0.04 (-0.16, -0.08)**
Flush	127 (5%)	19 (4%)	**0.43 (0.28-0.67)**	**-0.19 (-0.32, -0.07)**	**0.49 (0.28-0.84)**	**-0.12 (-0.26, -0.02)**
**Livestock owned[c]**						
None	1107 (47%)	250 (47%)	1.00	0.00	1.00	0.00
<5	463 (20%)	126 (24%)	1.13 (0.93-1.38)	0.02 (-0.06, 0.10)	1.07 (0.87-1.33)	0.01 (-0.08, 0.10)
>5	798 (34%)	159 (30%)	0.89 (0.74-1.06)	-0.06 (-0.13, 0.00)	0.90 (0.73-1.09)	-0.07 (-0.15, 0.00)
No of siblings	4 (3-6)	4 (3-6)	1.01 (0.98-1.05)	0.00 (-0.01, 0.01)	**0.95 (0.91-0.99)**	**-0.02 (-0.04, 0.00)**
Deceased father	67 (3%)	11 (3%)	1.08 (0.62-1.88)	-0.02 (-0.19, 0.16)	1.39 (0.78-2.47)	0.11 (-0.12, 0.34)

Significant associations are highlighted in bold. [a]Adjusted for child factors (age, sex, schooling and region of residence). [b]Adjusted for both child factors (age, sex, schooling, region of residence) and maternal factors (age, marital status, education level, economic/employment status, religion and ethnicity). [c]The test for linear trend for the levels of the factor was statistically significant. Associations for categorical variables (e.g. behavioural and emotional problems) are reported as risk ratios, whereas for continuous scores regression coefficients are used.

Supplementary figure 1. Histogram plots of the distribution of CBCL behavioural/emotional scores among the 3,273 preschool children.

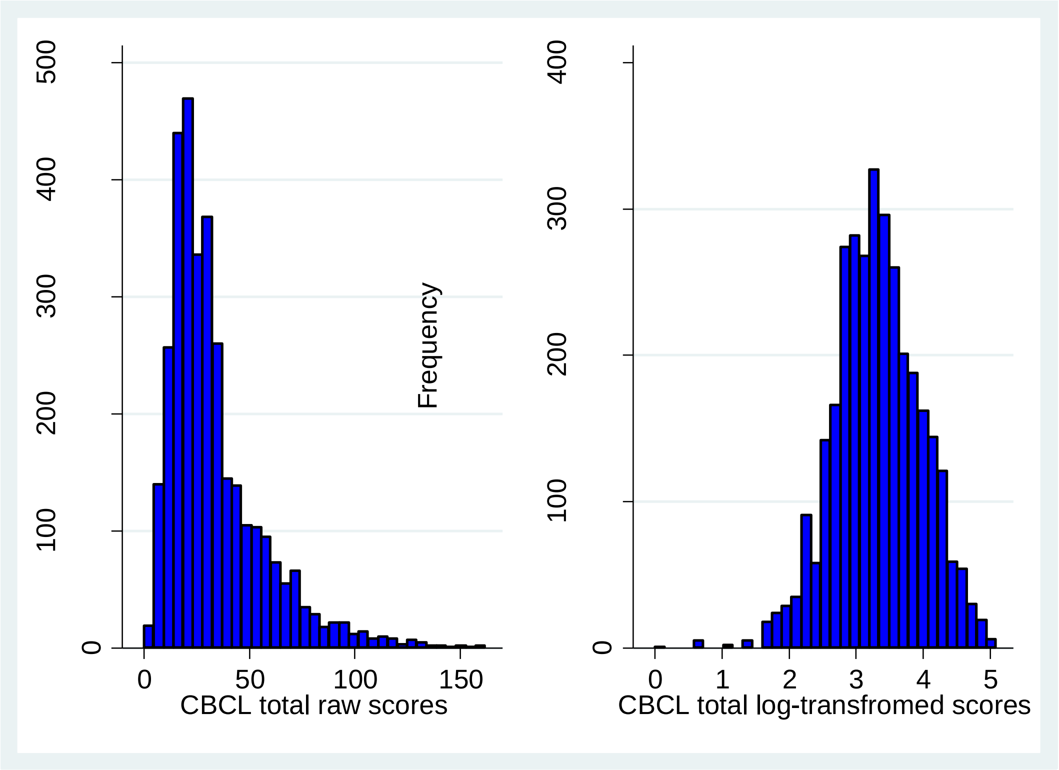

Skewed CBCL scores are shown on the left graph while the right graph shows the CBCL scores after log-transformation

Supplementary figure 2. Quintile plots of the distribution of CBCL behavioural/emotional scores among the 3,273 preschool children.

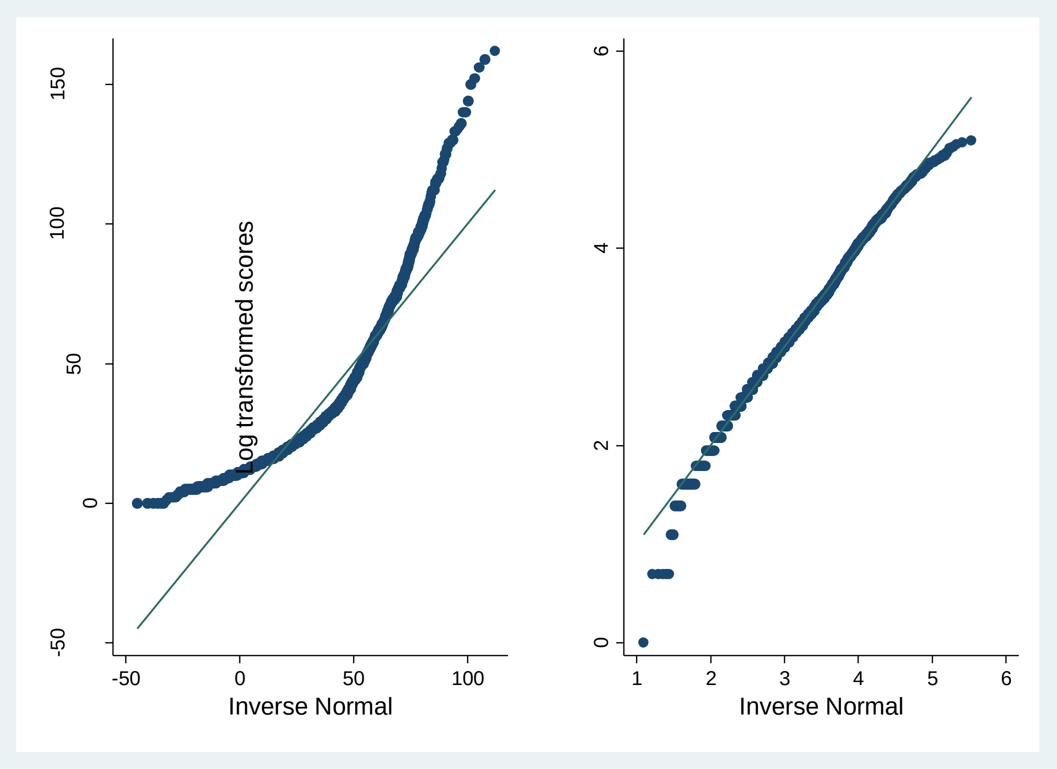

Supplementary figure 3. Kernel density plots of the distribution of CBCL behavioural/emotional scores among the 3,273 preschool children.

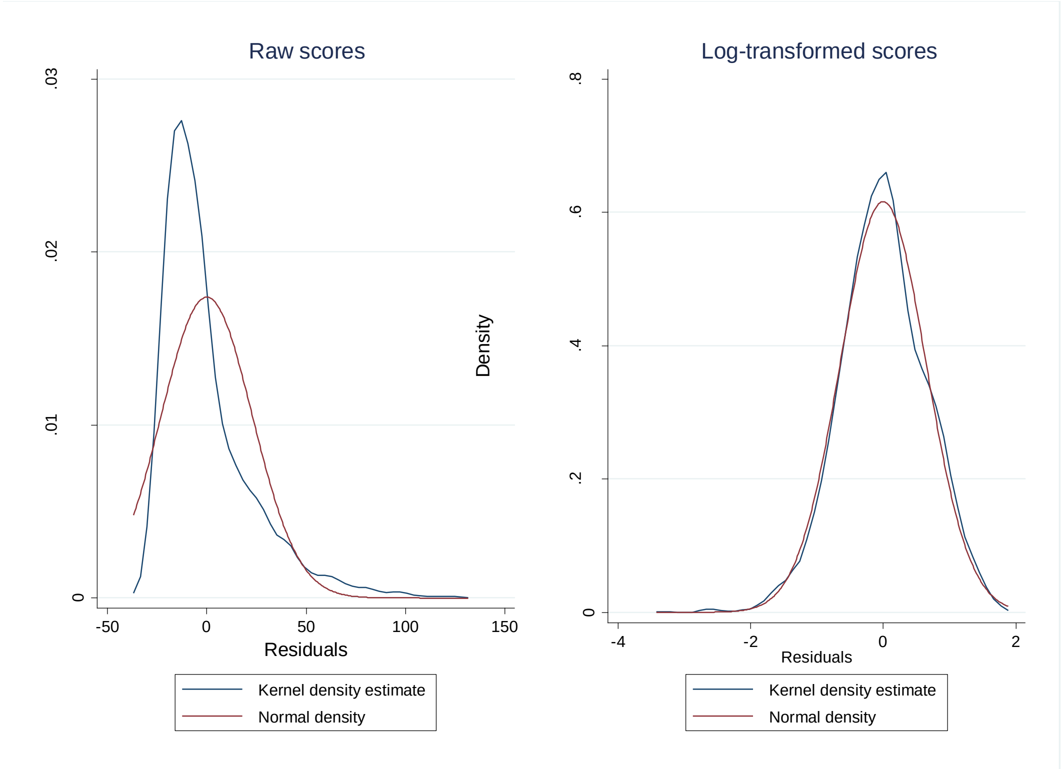

Supplement: Supplementary appendix [file mmc1.pdf]
